# Osseous implications of proton pump inhibitor therapy: An umbrella review

**DOI:** 10.1016/j.bonr.2024.101741

**Published:** 2024-02-01

**Authors:** Abdullah S. Alanazi, Hadiah Almutairi, Jeetendra Kumar Gupta, Dibyalochan Mohanty, Deepankar Rath, Ali A. AlOdan, Ahmed Mahal, Mahalaqua Nazli Khatib, Shilpa Gaidhane, Quazi Syed Zahiruddin, Sarvesh Rustagi, Prakasini Satapathy, Hashem Abu Serhan

**Affiliations:** a Department of Clinical Pharmacy, College of Pharmacy, Jouf University, Al-Jouf, Saudi Arabia; b Department of Pharmacy Practice, College of Pharmacy, University of Hafr Albatin, Saudi Arabia; c Institute of Pharmaceutical Research, GLA University Mathura, Uttar Pradesh, India; d Centre for Nano Medicine, Department of Pharmaceutics, School of Pharmacy, Anurag University, Hyderabad, India; e Department of Pharmacology, School of Pharmacy and Life Sciences, Centurion University of Technology and Management, Odisha, India; f Department of Family Medicine, Security Forces Hospital, Riyadh, Saudi Arabia; g Department of Medical Biochemical Analysis, College of Health Technology, Cihan University-Erbil, Erbil, Kurdistan Region, Iraq; h Division of Evidence Synthesis, Global Consortium of Public Health and Research, Datta Meghe Institute of Higher Education, Wardha, India; i One Health Centre (COHERD), Jawaharlal Nehru Medical College, Datta Meghe Institute of Higher Education, Wardha, India; j South Asia Infant Feeding Research Network (SAIFRN), Division of Evidence Synthesis, Global Consortium of Public Health and Research, Datta Meghe Institute of Higher Education, Wardha, India; k School of Applied and Life Sciences, Uttaranchal University, Dehradun, Uttarakhand, India; l Center for Global Health Research, Saveetha Medical College and Hospital, Saveetha Institute of Medical and Technical Sciences, Saveetha University, Chennai, India; m School of Pharmacy, Graphic Era Hill University, Dehradun, India; n Medical Laboratories Techniques Department, AL-Mustaqbal University, 51001 Hillah, Babil, Iraq; o Department of Ophthalmology, Hamad Medical Corporation, Doha, Qatar

**Keywords:** Proton pump inhibitors, Bone health, Fracture risk, Bone mineral density, Meta-analysis

## Abstract

**Background:**

Proton pump inhibitors (PPIs) are among the most commonly prescribed medications worldwide for acid-related disorders. While their short-term efficacy and safety are well-established, concerns regarding their long-term effects on bone health have emerged. This umbrella review aimed to synthesize the available findings on the associations between PPI use and bone metabolism outcomes.

**Methods:**

An electronic search was conducted using PubMed, Web of Science, Embase, and the Cochrane Database up to September 16, 2023. Systematic reviews and meta-analyses of randomized controlled trials (RCTs) and observational studies that evaluated the relationship between PPIs and bone metabolism outcomes were included. Data extraction, quality appraisal, and synthesis were performed in line with the Joanna Briggs Institute and PRISMA guidelines. The strength of the evidence was graded using the GRADE criteria. Statistical analysis was performed in R version 4.3.

**Results:**

Out of 299 records, 27 studies met the inclusion criteria. The evidence indicated a statistically significant increased risk of fractures, notably hip, spine, and wrist fractures, in PPI users. PPI use was associated with changes in Bone Mineral Density (BMD) across various bones, though the clinical relevance of these changes remains uncertain. Furthermore, PPI-induced hypomagnesemia, which can influence bone health, was identified. A notable finding was the increased risk of dental implant failures in PPI users. However, the certainty of most of the evidence ranged from very low to low based on GRADE criteria.

**Conclusion:**

The long-term use of PPIs may be associated with adverse bone health outcomes, including increased fracture risk, alterations in BMD, hypomagnesemia, and dental implant failure. While these findings highlight potential concerns for long-term PPI users, the current evidence's low certainty underscores the need for robust, high-quality research to clarify these associations.

## Graphical abstract

**Figure:**
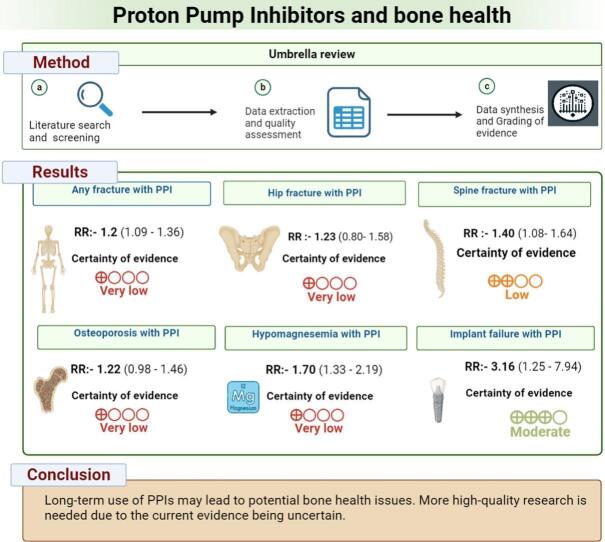

## 1 Introduction

Proton pump inhibitors (PPIs) are pivotal in modern medical protocols for treating disorders related to gastric acid, having emerged as one of the most frequently utilized medications worldwide. The surge in the usage of compounds like esomeprazole is largely attributed to the escalating occurrences of conditions like gastroesophageal reflux disease and peptic ulcers (Aguilera-Castro et al., 2016). PPIs operate by causing an irreversible inhibition of the hydrogen/potassium adenosine triphosphatase enzyme system (the H+/K+ ATPase, or the gastric proton pump) located in the gastric parietal cells, effectively limiting the secretion of gastric acid (Malfertheiner et al., 2017). The global reliance on PPIs is evidenced by substantial prescription trends, with instances like the 16 million prescriptions recorded in France in 2015 (Lespessailles and Toumi, 2022), and a noted escalation in prescribing prevalence in Germany between 2005 and 2013 (Hoffmann et al., 2014). Their status further broadens the reach of PPIs as over-the-counter medications, rendering them accessible to a broader demographic (Curtiss, 2002; Forgacs and Loganayagam, 2008; Sattayalertyanyong et al., 2020).

Nonetheless, the extensive consumption of PPIs has invoked concerns and prompted extensive investigations into their safety. Although they are integral in managing and preventing a variety of acid-related conditions, emerging evidence points towards a possible link between extended PPI consumption and a range of adverse health implications such as *clostridium difficile*-associated diarrhea, occurrence of community-acquired pneumonia, and potentially, an elevated risk of certain cancers through intestinal dysbiosis (Kwok et al., 2012; Lambert et al., 2015; Vaezi and Choksi, 2017). Moreover, concerns have been raised about the long-term impacts of PPIs on bone health (Poly et al., 2019; Paik et al., 2018; Zhou et al., 2016), and some recent studies have uncovered associations between the initiation of certain PPIs and increased occurrences of knee replacement surgeries (Zeng et al., 2022). The evidence suggests potential adverse impacts of PPIs on bone health and metabolism. These impacts include an increased risk of fractures, the development of osteoporosis, and a decrease in bone mineral density (Lespessailles and Toumi, 2022). Furthermore, the effect of PPIs on dental implant failure is also a subject of ongoing debate (Rogoszinski et al., 2022). A myriad of systematic reviews has been conducted, exploring the correlations between PPI use and various aspects of bone metabolism, including but not limited to, risk of fractures, onset of osteoporosis, and alterations in bone mineral density. A noticeable increase in systematic reviews on this topic has been discerned in recent years, highlighting growing concern and focus in the medical community on these potential correlations (Poly et al., 2019; Aleraij et al., 2020; da Maia et al., 2022).

An umbrella review synthesizes evidence from multiple systematic reviews on a specific topic, offering a comprehensive overview of the existing research (Aromataris et al., 2015). This study aims to conduct an umbrella review of existing research on PPIs affect bone metabolism. It will compile and analyze data to understand the varied impacts of PPIs on bone health and determine the relationship between PPI use and changes in bone metabolism.

## 2 Methods

This umbrella review was conducted as per the methodology described by the Joanna Briggs Institute (JBI) (Aromataris et al., 2014) and Preferred Reporting Items for Systematic Reviews and Meta-Analyses (PRISMA) guidelines (Table S1) (Page et al., 2021). The study is registered with PROSPERO under registration number: CRD42023465040.

### 2.1 Selection criteria

This umbrella review includes systematic reviews and meta-analyses of randomized controlled trials (RCTs) and observational studies that assessed the association between PPIs and bone metabolism and related outcomes such as fracture risk, bone mineral density changes, osteointegration of implants, hypomagnesemia, and osteoporosis. The following were excluded from this review: case reports, case series, animal studies, conference abstracts, and narrative reviews. Articles not available in English were also excluded. Refer to Table S2 for detailed inclusion criteria.

### 2.2 Literature search and screening

A literature search of the literature was undertaken in databases including Embase, PubMed, Web of Science, and the Cochrane Database to identify systematic reviews on the topic up to September 16, 2023. Keywords and MeSH terms related to “Proton pump inhibitors,” “systematic review,” and “meta-analysis” informed the search criteria. No restrictions were imposed on the publication year. The search strategy can be found in Table S3.

Two reviewers (PS, HA) independently assessed the search outcomes once duplicates had been removed via the Nested Knowledge software. The first level of screening focused on titles and abstracts, which was then followed by a comprehensive review of the full texts. Discrepancies in opinions about article inclusion were settled by seeking the input of a third reviewer (ASA).

### 2.3 Data extraction

Data extraction was performed by two reviewers (JKG, DM). They first extracted data from each eligible systematic review. Information such as author name, year of publication, databases and search year, objective of the study, type of participants, number and type of studies, risk of bias tools used and their results, outcomes of concern, effect size and confidence intervals (CI), *p* value, publication bias, and were obtained.

### 2.4 Quality appraisal

For assessing the quality of the included systematic reviews included in this study, JBI Checklist for Systematic Reviews and Research Syntheses was used (Aromataris et al., 2015). The JBI tool offers a comprehensive approach to appraise the quality of systematic reviews. It evaluates various aspects, including the clarity of the research question, the appropriateness of inclusion criteria, and the comprehensiveness of the search strategy, among others.

### 2.5 Data synthesis

The synthesis of evidence was presented in both narrative and tabular formats. We provided a table detailing the specifics of each systematic review included in our analysis. This encompassed information such as the number of primary studies and participants involved, outcomes assessed, and reported effect estimates, such as risk ratios (RR), odds ratios (OR), mean difference (MD), and Standardised mean difference (SMD). When available, their CIs, heterogeneity, publication bias, and final findings were also included. In addition, the table summarized the quality assessments and outlined the risk of bias identified in the primary studies. A narrative approach was employed to summarize the evidence for each outcome, complemented by tabular formats where applicable to ensure clarity. We prioritized the results of the systematic review rated highest by JBI tool.

A meta-analysis was conducted to determine pooled outcomes based on effect size such as RR, OR, and MD. We used a random effects model to pool results. The degree of heterogeneity among study findings was measured using I^2^ and tau-squared metrics (Langan et al., 2019). Both ranged from 0 % to 100 %, with higher values indicating greater inconsistency (Gandhi et al., 2023). A *p*-value below 0.05 was considered indicative of statistical significance. We calculated the tau-squared value using the maximum likelihood approach. The funnel plot was utilized to identify potential publication biases when >10 studies were available for each outcome. R software, version 4.3, was used for all statistical analyses (Shamim et al., 2023).

### 2.6 Certainty of evidence

The quality of evidence was determined using GRADE criteria (Grading of Recommendations, Assessment, Development, and Evaluations). Grading was performed by considering 5 domains, including risk of bias in the individual studies, inconsistency, indirectness, imprecision, and publication bias for each outcome (Langendam et al., 2013). We graded the strength of evidence as very low, low, moderate, or high (Table S4).

## 3 Results

### 3.1 Literature search

**Fig. 1:**
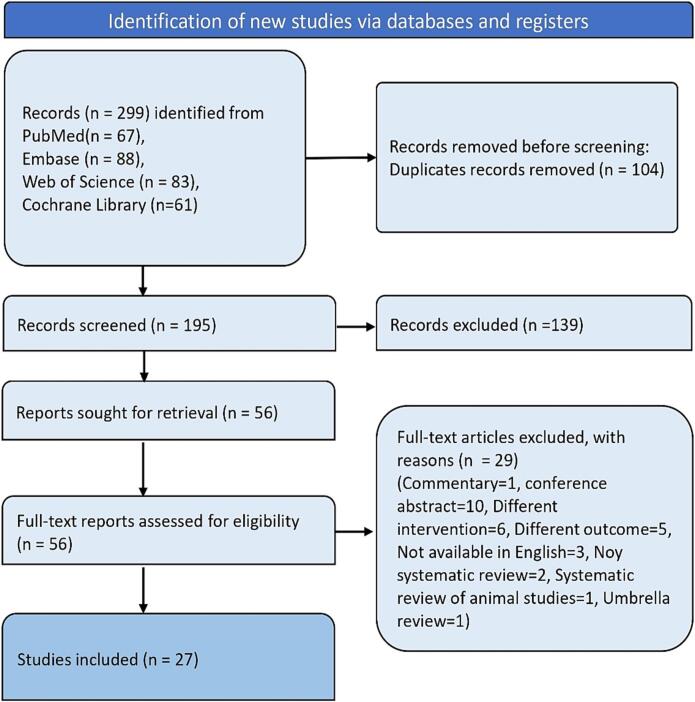
Flowchart showing screening and selection of articles.

A total of 299 records appeared in the database search from all databases, of which 104 were duplicates. Out of these, 195 records were screened, and 56 articles underwent a full-text eligibility check. 29 studies were excluded for various reasons, such as being conference abstracts, having the wrong intervention, wrong outcome, being commentaries, not being systematic reviews, being systematic reviews of animal studies, or being umbrella reviews. Ultimately, 27 studies met the criteria and were included in this review. Fig. 1 depicts the flow diagram of the screening and selection process.

### 3.2 Characteristics of included reviews

The important characteristics of included reviews are presented in Table 1**.** The systematic reviews focus on the association between PPIs and various bone-related outcomes, especially fracture risk, bone mineral density changes, and other drug-induced bone disorders. The research designs of the included studies ranged from prospective and retrospective cohort studies to case-control and nested case-control studies, with a few integrating cross-sectional and RCTs designs. These reviews covered diverse geographic locations, with a significant representation from the USA, UK, Canada, Denmark, and several European and Asian countries. The populations of interest varied from general patients to specific groups, such as menopausal women, children and young adults, hemodialysis patients, and patients undergoing dental implants. Predominantly, the outcomes of concern were related to fracture risk (hip, spine, wrist, and any fracture), bone mineral density (BMD) changes, dental implant failures, and other drug-related bone disorders. Risk of bias assessment tools, such as the Newcastle-Ottawa Scale (NOS), STROBE, Cochrane Adverse Effects Methods Group, NHLBI, and Cochrane RoB, were employed by different authors. The overall risk of bias in most studies ranged from low to moderate, although a few indicated high risk. Publication bias, when reported, was mainly assessed using techniques like Begg's test, Egger's test, and funnel plot asymmetry, with several studies showing no evidence of publication bias. The result of quality assessment is presented in Table S5.

**Table 1:** Characteristics of included reviews.

Study	Objective	Databases searched	Included Study designs	Number of studies	Country of included studies	Year of included studies	Population	Outcomes of concern	Risk of bias tool used	Overall risk of bias	Publication bias
Aggarwal 2019 (Aggarwal et al., 2019)	To consolidate the available data on drug induced bone disorders	PubMed, Medline, Embase (July 2019)	Prospective and Nested case-control	NA	NA	NA	NA	Risk of fracture, hip fracture	NA	NA	NA
Aghaloo 2019 (Aghaloo et al., 2019)	To evaluate the effect of systemic disorders, other diseases, and drugs on implant osseointegration	PubMed upto July 2018	Case control, prospective, retrospective studies	2 (for PPI)	NA	2001–2017	General	Implant survival rate	NA	NA	NA
Aleraij 2020 (Aleraij et al., 2020)	To evaluate the association between the use of PPIs and changes in bone mineral density	PubMed/MEDLINE, EMBASE, Cochrane, CINAHL Up to March 2019	Prospective and retrospective cohort studies	10	USA, the Republic of Kosovo, Canada, China, Turkey and South Korea	2008–2018	General	Mean annualized percent change in BMD, Mean difference in BMD	NOS	Not reported	NA
Cai 2015 (Cai et al., 2015)	To assess the relationship between use of antacid drugs and fracture risk	PubMed and Embase	Nested case control, case control, cohort	18	UK, USA, Spain, Canada, Netherlands, Europe, Sweden, Taiwan, Denmark	1997–2014	General	Hip fracture, any fracture, Spine fracture, wrist fracture	NA	NA	No publication bias
Chappuis 2018 (Chappuis et al., 2018)	To investigate the association between the intake of medications that may affect bone metabolism and implant outcomes	PubMed, MEDLINE (OVID), EMBASE (OVID), Cochrane Library, Web of Science, and SciVerse (Elsevier) up to May 2017	Retrospective cohort studies	2	Canada, Sweden	2017	Adults wearing implant-supported prostheses	Implant Failure	STROBE and NOS	Low risk of bias, Studies scored 8 and 9 on NOS	NA
Da Maia 2022 (da Maia et al., 2022)	To assess whether there is a relationship between the use of PPIs and fractures in menopausal women	PubMed, Scopus, and Science Direct, 12 April 2021	Observational prospective cohort studies	5	USA, Sweden, Germany, France, Australia	2009–2014	Menopausal women	Fractures	NOS	Overall NOS score 8–9, low risk of bias	NA
Eom 2011 (Eom et al., 2011)	To investigate the association between the use of PPIs or H2RAs and fracture risk	MEDLINE (PubMed), EMBASE, and the Cochrane Library from inception through December 2010	Case-control, nested case-control, cohort	10 (PPI)	USA, UK, Canada, Denmark, France, Taiwan, Netherlands	2006–2011 (for PPI)	General	Any Fractures, Hip fracture, spine fracture, wrist fracture	NOS	moderate to high	No publication bias detected (Egger, *p* = 0.45)
Fan 2017 (Fan et al., 2017)	To evaluate the association between PPI use and risk of osteoporosis	PubMed, EMBASE and Web of Knowledge from inception up to March 2017.	Case-control, prospective studies	27	China, Germany, Taiwan, Australia, UK, USA, Korea, Denmark, Spain, Canada, Europe, Netherlands	2006–2016	General patient population	Osteoporosis, any Fracture, Hip fracture, Spine fracture	NOS	Moderate to high	No publication bias detected
Heidelbaugh2009 (Heidelbaugh et al., 2009)	To summarize adverse risks associated with long-term use of PPIs in the treatment of upper gastrointestinal disorders	MEDLINE (1966–2008)	Nested case-control, Case-control, Retrospective matched cohort	3	UK, Canada, Denmark	2006 and 2008	General Practice database from UK, Community based from Denmark and Canada	Bone fracture	NA	NA	NA
Hussain 2018 (Hussain et al., 2018)	Tor explore the association of PPI use and risk of hip fracture	MEDLINE via PubMed and Cochrane central	Cohort, Case control and nested case-control	17	USA, UK, Canada, Taiwan, Spain, Netherlands	2006 to 2017	Patients with PPI exposure	Hip fracture	NOS	Medium to high quality	NA
Islam 2018 (Islam et al., 2018)	To quantify the associations as presented in the literature and to also provide this information to healthcare professionals and patients about PPIs potentially adverse effects	Medline (PubMed), Embase, and the Cochrane Library (July 2016)	Case-control, cohort, and cross-sectional	43 (12 studies on Hip fracture)	USA, UK, Spain, Taiwan, Korea, Netherlands		General	Hip fracture	Modified NOS	NA	NA
Kwok 2011 (Kwok et al., 2011)	To perform a meta-analysis of fractures in patients taking PPIs and H2RAs	MEDLINE and Embase September 2010	Case-control, prospective and retrospective cohort	12	USA, UK, Netherlands, Europe, Canada	1997–2010	General	Spine fracture, hip fracture, overall fracture	Cochrane Adverse Effects Methods Group	Unclear and low risk of biases	NA
Li 2021 (Li et al., 2021)	To evaluate the risk of fracture with PPIs and H2RAs use in children and young adults	PubMed, EMBASE database, Cochrane Library, and Web of Science (May 2021)	Retrospective cohort, cohort, case control	6	Sweden, Israel, UK, USA	2015–2020	Children and young adults	Risk of fracture	NOS	High	No publication bias detected
Liu 2019 (Liu et al., 2019)	To determine the link between PPI use and fractures, osteoporosis, and BMD loss	PubMed, EMBASE and the Cochrane Library from inception up to May 2018	Case-control, Cohort and cross-sectional studies	32	USA, Canada, UK, France, Denmark, Netherland, Iran	2006–2018	General	Fracture/Osteoporosis/ Bone mineral loss	NOS	All studies scored moderate to high score for NOS	No publication bias detected for hip, any fracture, and osteoporosis (*p* = 0.54, 0.39, 0.07), Spine fracture showed publication bias (*p* = 0.03)
Mortensen 2020 (Mortensen et al., 2020)	To assess the impact of various classes of medications on the risk of fragility hip fracture	EMBASE, PubMed, Web of Science, and Cochrane Central and clinicaltrials.gov in September 2017.	NA	38	Denmark, USA, Norway, Netherlands, UK, Taiwan, Ireland, France, Greece, Canada, Austria, Germany	1981 to 2017,	General	Hip fractures	NOS	High quality	No publication bias detected, Begg and Mazumdar test for rank correlation (*p* = 0.35)
Nassar 2018 (Nassar and Richter, 2018)	To evaluate the relation-ship between PPI use and fracture incidence	PubMed, Embase, and Google Scholar, Feb 2018	Prospective or retrospective observational studies	33	NA	2006–2017	General and patient population	Fracture risk, Change in BMD	NA	NA	No publication bias detected. Begg's test (*p* = 0.15)
Ngamruengphong 2011 (Ngamruengphong et al., 2011)	To evaluate an association between the use of PPI and risks for fracture	MEDLINE, EMBASE, and Cochrane Up to Aug 2010	Cohort and Case-control studies	10	UK, Denmark, USA, Netherlands, Canada, Europe	2006–2010	Men and women aged >50 years, Men and women aged >43 years, Men and women aged >18 years, Men and women aged 50–79 years, Post-menopausal women	Hip fracture, Spine, wrist, Any fracture	Validity criteria suggested by Loke et al. (9) and Levine (10)	Overall Low-moderate Risk of bias, only one study had high bias	NA
Poly 2019 (Poly et al., 2019)	To gauge precisely the nature and magnitude of the association between PPIs and hip fracture risk	PubMed, EMBASE, Scopus, Google Scholar, and Web of Science (January 1990 and March 2018)	Cohort and case-control studies	24	USA, UK, Taiwan, Denmark, Korea, Netherlands, Canada, Finaland	2006 to 2018	Adults (aged 18 years or greater)	Development of hip fracture	NOS	Moderate to high	Eggerâ€™s regression test of the funnel asymmetry showed no observed significantpublication bias (*p* value = 0.75).
Srinutta 2019 (Srinutta et al., 2019)	To find out an association between PPI dose or treatment duration and the development of hypomagnesemia	MEDLINE, Scopus, Cochrane (1978 to June 2018)	Cross-sectional, case-control, cohort studies	16	North America = 7, Europe = 6, Asia = 3	2012–2018	Patients in ambulatory settings, dialysis facilities, hospital settings	Hypomagnesemia	NHLBI	Fair to good	Not present Egger p value = 0.69
Verma 2022 (Verma, 2022)	To determine the influence of PPIs on biomechanical efficiency of dental implants	PubMed, Cochrane database, EBSCO host, Web of Science and Scopus from 2010 upto Dec 2021	RCTs	6	NA	2017–2019 (human)	Patients undergoing dental implant treatment modality	Dental implant failure	Cochrane RoB	Low bias	NA
Vestergaard2020 (Vestergaard, 2020)	To perform systematic review of drugs inducing bone loss or associated with fracture risk	Medline	NA	5	NA	2006–2019	General	Bone loss	NA	NA	NA
Vinnakota 2020 (Vinnakota and Kamatham, 2020)	To find out the usage of PPIs in individuals undergoing dental implantation influence the success of an implant compared to controls	MEDLINE, Ovid and Cochrane Up to July 2019	Retrospective cohort studies	3	Canada, Turkey, Sweden	2017–2019	Patients who underwent dental implant	Dental implant failure	NOS	Low risk of bias, good quality overall	NA
Yang 2022 (Yang et al., 2022)	To evaluate the risk of fracture in children and young adults exposed to ASDs	Cochrane Library, PubMed, and EMBASE (inception to December 2020)	Cohort, Nested case-control study	6	USA, UK, Israel, Sweden	2015 to 2020	Children, Young adults	Fracture risk	NOS	Four studies out of 6 scored high quality	NA
Ye 2011 (Ye et al., 2011)	To determine whether the association between PPIs and hip fracture exists quantitatively	PubMed and EMBASE, Cochrane up to June 2010	Prospective and retrospective cohort studies, case-control studies	7	Netherlands, USA, UK, CANADA, Denmark	2008–2011	18 and older, 50–79 aged, post-menopausal women	Hip fracture	STROBE	Not reported	No publication bias detected
Yu 2011 (Elaine et al., 2011)	To estimate the overall effect of PPI use on fracture rates	PubMed/MEDLINE, EMBASE, Web of Science, and BIOSIS Previews upto October 10, 2010	Case-control, retrospective longitudinal cohort	11	USA, UK, Denmark, Europe, Netherlands, Taiwan	2006–2010	Adults (Predominantly postmenopausal women and older men)	Hip fracture, Any fracture, Spine fracture	NA	NA	No evidence of publication bias (Begg's test *p* = 0.22)
Zhang 2022 (Zhang et al., 2022)	To find put the impact of PPIs on Hemodialysis patient outcomes	Pubmed, Embase, Cochrane Library, and Web of Science, April 2022	Prospective, Retrospective and cross-sectional studies	12	Japan, USA, Croatia, Denmark, Multicentre, Spain	2013–2019	Hemodialysis Patients	Fracture, Hypomagnesemia, Vascular calcification	NOS	Moderate to high	No publication bias detected
Zhou 2016 (Zhou et al., 2016)	To further clarify the association between PPI use and fracture risk	PubMed upto Feb 2015	Cohort and case-control studies	17	UK, Denmark, Canada, USA, Europe, Sweden, Spain, Netherlands, Taiwan, Australia	1987–2012	General	Hip fracture, Spine and all site fracture	NOS	Good quality	No publication bias detected for any site fracture (*p* = 0.297), Detected for spine fracture (*p* = 0.038)

Abbreviations: - ASDs - Acid Suppressing Drugs, BMD - Bone Mineral Density, H2RAs - Histamine-2 Receptor Antagonists, MEDLINE - Medical Literature Analysis and Retrieval System Online, NOS - Newcastle-Ottawa Scale, NHLBI - National Heart, Lung, and Blood Institute, PPI - Proton Pump Inhibitor, RCTs - Randomized Controlled Trials, RoB - Risk of Bias, STROBE - Strengthening The Reporting of Observational studies in Epidemiology.

### 3.3 Summary of outcomes

Table 2 presents the overall summary of results based on each outcome.

**Table 2:** Summary of outcomes.

Outcome	Number of studies	Type of effect estimate	Effect size	95 % CI	Heterogeneity (I^2^)	Publication bias	Grade
Any fracture	14	RR	1.2	1.09–1.36	85 %	No	Very Low
Fracture risk (children)	5	RR	1.12	1.07–1.17	18 %	NA	Low
Fracture risk (young adults)	2	RR	0.98	0.31–1.65	84 %	NA	Very Low
Hip fracture	26	RR	1.2	1.13–1.27	64 %	No	Very Low
Hip fracture (post-menopausal women)	5	RR	1.23	0.8–1.58	30 %	NA	Very Low
Spine fracture	6	RR	1.4	1.18–1.64	36 %	NA	Low
Wrist fracture	3	RR	1.08	0.7–1.44	62 %	NA	Very Low
Osteoporosis	6	RR	1.22	0.98–1.46	92 %	NA	Very Low
BMD (all)	7	MD	0.025	0.001–0.050	55 %	NA	Very Low
BMD (femur)	5	MD	−0.094	−0.409- 0.022	72 %	NA	Very Low
BMD (spine)	7	MD	0.025	−0.047-0.097	48 %	NA	Very Low
BMD (hip)	4	MD	0.018	−0.030- 0.066	54 %	NA	Very Low
Hypomagnesemia	12	OR	1.7	1.33–2.19	88 %	No	Very Low
Implant failure	4	RR	3.16	1.25–7.94	96 %	NA	Moderate
Bone fracture (hemodialysis patients)	3	OR	1.29	1.21–1.37	0 %	No	Low
Hip fracture (hemodialysis patients)	3	OR	1.37	1.12–1.67	82 %	No	Very low
Hypomagnesemia (hemodialysis patients)	4	OR	2.79	1.95–4.00	0 %	No	Moderate
Aortic calcifications (hemodialysis patients)	2	OR	2.03	1.28–3.24	0 %	NA	Low

#### 3.3.1 Fracture risk

**Fig. 2:**
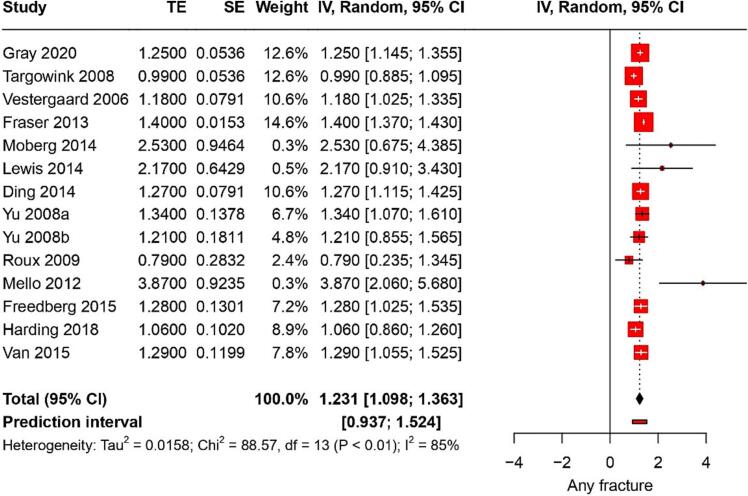
Forest plot showing the pooled result of PPI use and risk of any fracture.

**Fig. 3:**
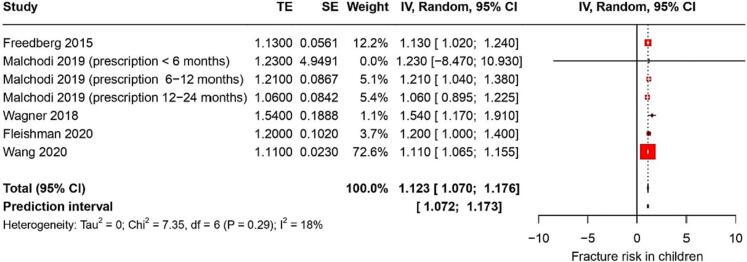
Forest plot showing the pooled result of PPI use and fracture risk in children.

Fourteen studies reported on fracture risk. They identified a pooled RR of 1.2 (95 % CI: 1.09–1.36) with a heterogeneity of 85 % (Fig. 2). The certainty of this evidence was very low. In children, PPI use was associated with a RR of hip fractures of 1.12 (95 % CI: 1.07–1.17) (Fig. 3); the certainty of this evidence was low. Among young adults, PPI use was linked to a RR of hip fractures of 0.98 (95 % CI: 0.31–1.6); the certainty of this evidence was also very low.

#### 3.3.2 Hip fracture

**Fig. 4:**
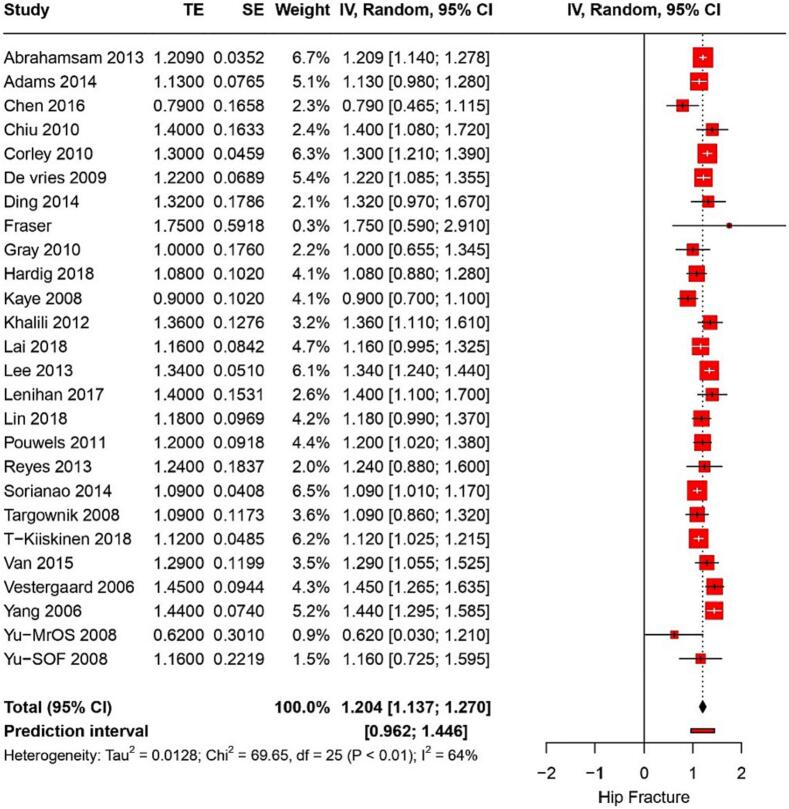
Forest plot showing the pooled result of PPI use and hip fracture.

**Fig. 5:**
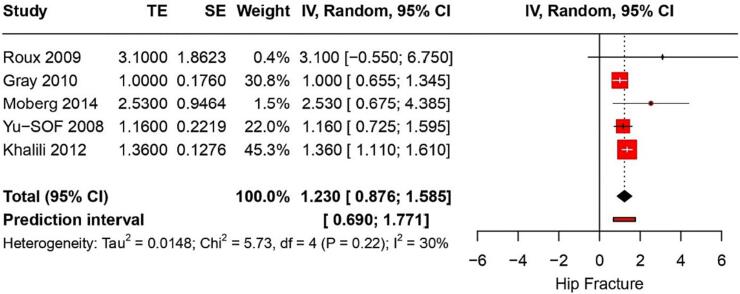
Forest plot showing the pooled result of PPI use and hip fracture risk in postmenopausal women.

From 26 studies, the risk of hip fracture associated with PPI use was found to have a RR of 1.2 (95 % CI: 1.13–1.27) with a heterogeneity of I^2^ = 64 % (Fig. 4). The certainty of this evidence was very low. Five studies on postmenopausal women showed that PPI use was linked to a RR of hip fractures of 1.2 (95%CI: 0.87–1.5) (*p* < 0.006) (Fig. 5). The certainty of this evidence was very low. Furthermore, three studies reported hip fractures in hemodialysis patients with a pooled OR of 1.37 (95 % CI: 1.12–1.67) and I^2^ of 82 %; the evidence's certainty was very low.

#### 3.3.3 Spine fracture

Six studies showed that the risk of spine fracture associated with PPI use had a RR of 1.4 (95 % CI: 1.18–1.64) with a heterogeneity of I^2^ = 36 %. The certainty of this evidence was very low.

#### 3.3.4 Wrist fracture

From three studies, the risk of wrist fracture linked to PPI use was determined to have a RR of 1.08 (95 % CI: 0.71–1.44) with a heterogeneity of I^2^ = 62 %. The certainty of this evidence was very low.

#### 3.3.5 Osteoporosis

**Fig. 6:**
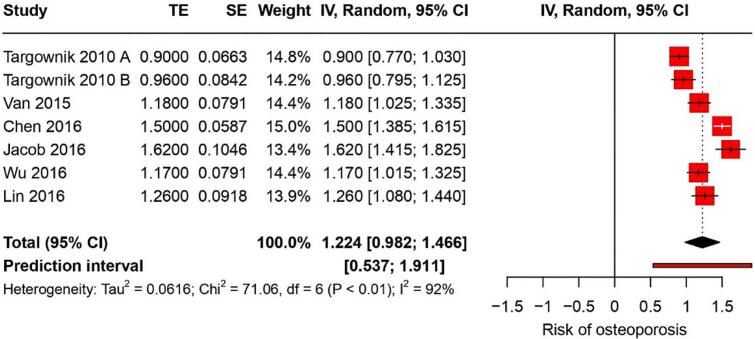
Forest plot showing the pooled result of PPI use and risk of osteoporosis.

Six studies indicated that the risk of osteoporosis associated with PPI use had a RR of 1.22 (95 % CI: 0.98–1.46) with a high heterogeneity of I^2^ = 92 % (Fig. 6). The certainty of this evidence was very low.

#### 3.3.6 BMD

**Fig. 7:**
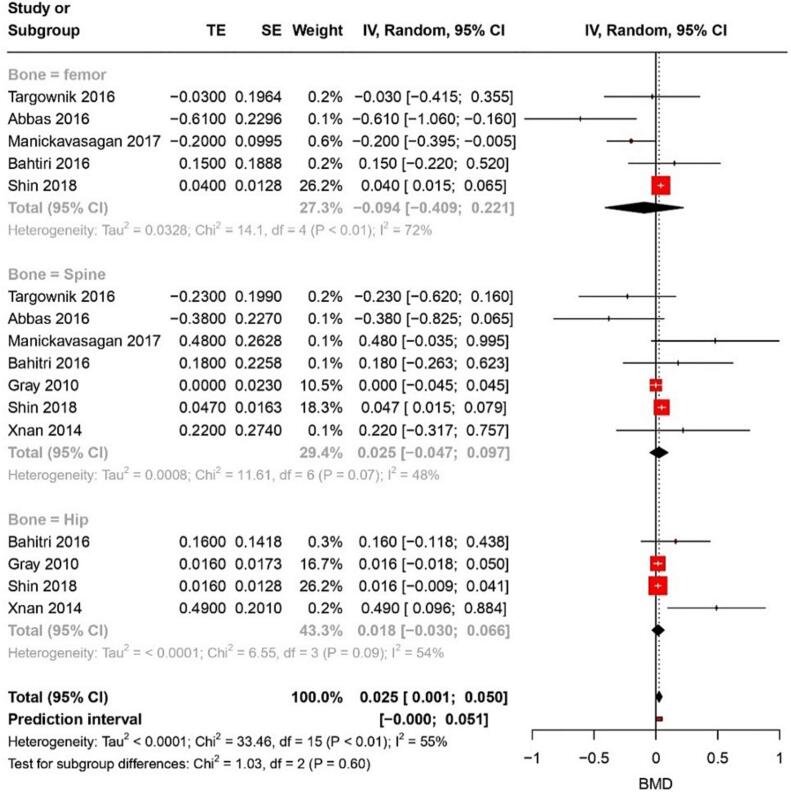
Forest plot showing the pooled result of PPI use and change in BMD.

Seven studies reported a MD in BMD of 0.025 (95 % CI: 0.001–0.50) with an I^2^ of 55 % for all bone types combined (Fig. 7). The certainty of this evidence was very low. For femur BMD, five studies showed an MD of −0.094 (95 % CI: 0.409–0.22) with 48 % heterogeneity. Meanwhile, for the spine, five studies revealed an MD for BMD of 0.025 (95 % CI: 0.047–0.097). Lastly, for the hip bone, an MD of 0.018 (95 % CI: −0.030–0.66) was found from four studies with an I^2^ of 54 %.

#### 3.3.7 Hypomagnesemia

Twelve studies reported hypomagnesemia in the general population with PPI use, resulting in an OR of 1.7 (95 % CI: 1.33–2.19) and an I^2^ of 88 %. The certainty of this evidence was very low. In hemodynamic patients, PPI use led to an OR of 2.27 (95 % CI: 1.95–4.00) from four studies for hypomagnesemia, with the certainty of this evidence being moderate.

#### 3.3.8 Implant failure

Four studies identified a RR of 3.15 (95 % CI: 1.25–7.94) for dental implant failure associated with PPI use. The heterogeneity was high with an I^2^ of 96 %. The certainty of this evidence was moderate.

## 4 Discussion

Among the most widely prescribed medications worldwide for the treatment of acid-related disorders are PPIs. While these medications are generally considered safe for short-term use, concerns regarding their long-term effects on bone health have been emerging. This umbrella review synthesized the available evidence from systematic reviews and meta-analyses to provide a comprehensive understanding of the associations between PPI use and alterations in bone metabolism. We could cover almost all outcomes related to bone metabolism.

Our findings highlight several associations of PPI use with bone-related outcomes. Predominantly, the evidence indicates a statistically significant, albeit modest, increased risk of fractures, including hip, spine, and wrist fractures, in individuals on PPIs. Notably, the fracture risk was found to be more pronounced in specific populations like children and post-menopausal women. These findings corroborate the concerns raised in earlier studies regarding the potential deleterious effects of acid suppression on bone health (Lespessailles and Toumi, 2022; Yu et al., 2008). The precise mechanisms underpinning this increased risk remain uncertain. However, it is postulated that long-term PPI use might interfere with calcium absorption due to the reduced stomach acid, thereby weakening bone strength (Ito and Jensen, 2010). Additionally, interference with osteoclast function, leading to altered bone remodelling, may play a role (Krüger et al., 2021). Besides fractures, our review identified a potential link between changes in BMD and PPI use. Although the mean differences in BMD across various bones were statistically significant in some studies, the clinical significance of these changes remains uncertain and necessitates further elucidation. It is worth noting that BMD is a crucial predictor of fracture risk, and even marginal reductions can culminate in clinically meaningful increases in fracture risk over time (Cefalu, 2004). Another most discussed is hypomagnesemia, a condition where blood magnesium levels are significantly reduced. Magnesium is not just an essential electrolyte for various physiological functions, but it also plays a pivotal role in bone health. Magnesium contributes to bone mineral density, serving as a cofactor in the enzymes that help deposit calcium into the bones. The etiology of PPI-induced hypomagnesemia is thought to be multifactorial, encompassing reduced intestinal absorption and increased renal magnesium wasting. A deficiency in magnesium can disrupt this balance, leading to weakened bones and an increased risk of fractures. Furthermore, magnesium deficiency has been linked to osteoporosis (Castiglioni et al., 2013). Given that PPI-induced hypomagnesemia might result in reduced magnesium availability for bone metabolism, the long-term use of these drugs could indirectly influence bone health. Interestingly, our review synthesized findings on a substantial risk of dental implant failure in PPI users. This underscores the broader implications of PPIs on skeletal health beyond the traditionally assessed outcomes. The underlying mechanisms for this observed association remain speculative but could be linked to altered bone metabolism and healing processes in PPI users (Rogoszinski et al., 2022). While there was a notable association between PPI use and outcomes related to bone health, the reliability of the existing evidence was deemed to be either very low or low for the majority of these outcomes as per the GRADE criteria. Such low certainty suggests that future research might change the estimates and our understanding. Several factors contribute to this uncertainty, including the high heterogeneity observed across the included reviews, and variations in study populations and designs. The extensive heterogeneity, in particular, makes interpretation challenging, as it hints at potential differences in study methodologies, populations, or both.

The adverse effects of PPIs are not limited to bone metabolism. For instance, A prior umbrella review highlighted a relation between the use of PPIs and various negative health effects, including those related to COVID-19, other infections, cardiovascular issues, bone-related complications, cancer, neurological, and renal problems (Veettil et al., 2022). In addition, another umbrella review explored the connection between PPI use and major adverse cardiovascular events (MACE), encompassing myocardial infarction, stroke and overall mortality (Teperikidis et al., 2023). The conclusions from these individual studies varied, with some indicating a direct relationship between MACE and PPI use, some finding no correlation, and some presenting inconclusive findings. Notably, a significant portion of the observational studies suggested a direct relation between PPI use and MACE.

Our findings together with previous reviews, highlight the importance of re-evaluating the risk-benefit profile of prescribing PPIs, particularly for extended durations (more than a year). The modest increase in fracture risk we identified, while statistically significant, carries different implications in clinical practice, depending on the risk factors of individual patients. These results should be interpreted within the context of each patient's overall fracture risk profileThe changes in BMD linked to PPI use, though statistically significant in some studies, may not lead to immediate clinical concerns. However, it's crucial to acknowledge that even minor reductions in BMD could, over time, result in a cumulative increase in fracture risks. Clinicians should be aware of the potential ramifications of PPIs on bone health, especially given the associations with increased fracture risks in vulnerable groups such as children and post-menopausal women, where even small decreases in bone density can have a significant impact on long-term bone health. It is crucial to weigh the therapeutic advantages against potential risks. PPIs, widely recognized for their efficacy in treating acid-related disorders, require careful consideration to ensure that the benefits of treatment adequately outweigh the risks. Tailored approaches and periodic re-assessment of PPI therapy are essential in managing the delicate balance between effective acid suppression and maintaining optimal bone health.

Periodic assessments of bone mineral density and serum magnesium levels in prolonged PPI users might be useful for early detection and intervention. The observed correlation between PPI use and dental implant failure adds another layer to clinical decision-making, necessitating additional counseling or alternative therapies for those undergoing dental procedures. Beyond bone health, the links between PPI use and other adverse health effects, including cardiovascular issues (Ariel and Cooke, 2019; Geng et al., 2023), underscore the need for a comprehensive approach in assessing the appropriateness of PPI therapy. A judicious and periodic re-evaluation of the necessity of ongoing PPI treatment, bearing in mind the potential cumulative risks, is advisable (Zhai et al., 2022).

Future research in this area should prioritize long-term, prospective studies to more clearly determine the causal relationship between PPI use and bone health outcomes. These studies need to encompass a range of diverse population groups and explore variations in PPI dosages and treatment durations. Additionally, there is a significant need for mechanistic studies to explore into how PPIs influence bone metabolism at the molecular and cellular levels, which could lead to new preventive strategies or alternative treatments. Complementing this, the use of real-world data in assessing the impact of PPIs across different clinical scenarios will offer a more comprehensive view of their effects on bone health. This approach will be particularly valuable in pinpointing patient groups more susceptible to adverse bone outcomes related to PPI use.

Our umbrella review has several strengths. Firstly, we undertook a comprehensive examination of all outcomes related to bone health associated with PPI use. Secondly, we employed the GRADE approach to assess the certainty of evidence, which ensures a systematic and rigorous evaluation of the available data. However, our review is not without limitations. As is inherent with all secondary research, our conclusions are bound by the quality and comprehensiveness of the primary systematic reviews we sourced. Moreover, the notable heterogeneity among the included studies points to potential variations in study design, participant populations, or both. These variations might raise concerns about the generalizability of our findings.

## 5 Conclusion

We could elucidate several potential adverse effects of PPIs on bone health, including increased risks of fractures, altered BMD, hypomagnesemia, and dental implant failure. These findings underscore the importance of the judicious use of PPIs, considering the potential risks against the benefits. Clinicians should be vigilant about the prolonged use of PPIs, especially in populations with already heightened fracture risks. Additionally, patients on long-term PPI therapy might benefit from regular bone health assessments. As the certainty of evidence for most outcomes remains low, further high-quality primary studies are essential to bolster our understanding of these associations and inform clinical practice more definitively.

## Funding

This study received no funding.

## CRediT authorship contribution statement

**Abdullah S. Alanazi:** Methodology, Investigation, Data curation, Conceptualization. **Hadiah Almutairi:** Project administration, Methodology, Investigation, Formal analysis. **Jeetendra Kumar Gupta:** Software, Resources, Project administration, Data curation, Conceptualization. **Dibyalochan Mohanty:** Validation, Supervision, Software, Investigation, Conceptualization. **Deepankar Rath:** Writing – review & editing, Investigation, Data curation. **Ali A. AlOdan:** Writing – review & editing, Supervision, Methodology, Data curation. **Ahmed Mahal:** Writing – review & editing, Supervision, Resources, Methodology. **Mahalaqua Nazli Khatib:** Writing – review & editing, Writing – original draft, Supervision, Software, Resources, Investigation, Formal analysis, Conceptualization. **Shilpa Gaidhane:** Writing – original draft, Visualization, Validation, Supervision, Data curation. **Quazi Syed Zahiruddin:** Software, Resources, Data curation, Conceptualization. **Sarvesh Rustagi:** Writing – review & editing, Supervision, Resources, Conceptualization. **Prakasini Satapathy:** Investigation, Formal analysis, Data curation, Conceptualization. **Hashem Abu Serhan:** Writing – review & editing, Formal analysis, Conceptualization.

## Declaration of competing interest

The authors declare that they have no known competing financial interests or personal relationships that could have appeared to influence the work reported in this paper.

## Data Availability

Data will be made available on request.

## Appendix A Supplementary data

Supplementary material Image 1
