# Supplementary material for: Osseous implications of proton pump inhibitor therapy: An umbrella review
Source: Bone Rep. 2024 Feb 1;20:101741. doi: 10.1016/j.bonr.2024.101741 (PMC10859261; doi:10.1016/j.bonr.2024.101741)
Supplement: Supplementary file 1 — Supplementary material [file mmc1.docx]

**SUPPLEMENTARY MATERIALS**

**Table S1.** PRISMA Checklist

| **Section and Topic** | **Item #** | **Checklist item (Prevalence of kidney diseases among the dengue patients: A systematic review and meta-analysis)** | **Location where item is reported** |
| --- | --- | --- | --- |
| **TITLE** | | |  |
| Title | 1 | Identify the report as a systematic review. | 1 |
| **ABSTRACT** | | |  |
| Abstract | 2 | See the PRISMA 2020 for Abstracts checklist. (made as per the Journal guidelines) | 2 |
| **INTRODUCTION** | | |  |
| Rationale | 3 | Describe the rationale for the review in the context of existing knowledge. | 3 |
| Objectives | 4 | Provide an explicit statement of the objective(s) or question(s) the review addresses. | 3 |
| **METHODS** | | |  |
| Eligibility criteria | 5 | Specify the inclusion and exclusion criteria for the review and how studies were grouped for the syntheses. | Table S2 |
| Information sources | 6 | Specify all databases, registers, websites, organisations, reference lists and other sources searched or consulted to identify studies. Specify the date when each source was last searched or consulted. | 4 |
| Search strategy | 7 | Present the full search strategies for all databases, registers and websites, including any filters and limits used. | Table S3 |
| Selection process | 8 | Specify the methods used to decide whether a study met the inclusion criteria of the review, including how many reviewers screened each record and each report retrieved, whether they worked independently, and if applicable, details of automation tools used in the process. | 4 |
| Data collection process | 9 | Specify the methods used to collect data from reports, including how many reviewers collected data from each report, whether they worked independently, any processes for obtaining or confirming data from study investigators, and if applicable, details of automation tools used in the process. | 4 |
| Data items | 10a | List and define all outcomes for which data were sought. Specify whether all results that were compatible with each outcome domain in each study were sought (e.g., for all measures, time points, analyses), and if not, the methods used to decide which results to collect. | 4, |
|  | 10b | List and define all other variables for which data were sought (e.g., participant and intervention characteristics, funding sources). Describe any assumptions made about any missing or unclear information. | 4 |
| Study risk of bias assessment | 11 | Specify the methods used to assess risk of bias in the included studies, including details of the tool(s) used, how many reviewers assessed each study and whether they worked independently, and if applicable, details of automation tools used in the process. | 4, |
| Effect measures | 12 | Specify for each outcome the effect measure(s) (e.g. risk ratio, mean difference) used in the synthesis or presentation of results. | NA |
| Synthesis methods | 13a | Describe the processes used to decide which studies were eligible for each synthesis (e.g. tabulating the study intervention characteristics and comparing against the planned groups for each synthesis (item #5)). | 4, 5 |
|  | 13b | Describe any methods required to prepare the data for presentation or synthesis, such as handling of missing summary statistics, or data conversions. | NA |
|  | 13c | Describe any methods used to tabulate or visually display results of individual studies and syntheses. | 4 |
|  | 13d | Describe any methods used to synthesize results and provide a rationale for the choice(s). If meta-analysis was performed, describe the model(s), method(s) to identify the presence and extent of statistical heterogeneity, and software package(s) used. | 5 |
|  | 13e | Describe any methods used to explore possible causes of heterogeneity among study results (e.g. subgroup analysis, meta-regression). | 5 |
|  | 13f | Describe any sensitivity analyses conducted to assess robustness of the synthesized results. | 5 |
| Reporting bias assessment | 14 | Describe any methods used to assess risk of bias due to missing results in a synthesis (arising from reporting biases). | NA |
| Certainty assessment | 15 | Describe any methods used to assess certainty (or confidence) in the body of evidence for an outcome. | 5 |
| **RESULTS** | | |  |
| Study selection | 16a | Describe the results of the search and selection process, from the number of records identified in the search to the number of studies included in the review, ideally using a flow diagram. | 5, |
|  | 16b | Cite studies that might appear to meet the inclusion criteria, but which were excluded, and explain why they were excluded. | 5, Table 1 |
| Study characteristics | 17 | Cite each included study and present its characteristics. | Table-1 |
| Risk of bias in studies | 18 | Present assessments of risk of bias for each included study. | Table S5 |
| Results of individual studies | 19 | For all outcomes, present, for each study: (a) summary statistics for each group (where appropriate) and (b) an effect estimate and its precision (e.g. confidence/credible interval), ideally using structured tables or plots. | Table 1, Table 2 |
| Results of syntheses | 20a | For each synthesis, briefly summarise the characteristics and risk of bias among contributing studies. | 5 |
|  | 20b | Present results of all statistical syntheses conducted. If meta-analysis was done, present for each the summary estimate and its precision (e.g. confidence/credible interval) and measures of statistical heterogeneity. If comparing groups, describe the direction of the effect. | 5, 6 |
|  | 20c | Present results of all investigations of possible causes of heterogeneity among study results. | 6,7 |
|  | 20d | Present results of all sensitivity analyses conducted to assess the robustness of the synthesized results. | NA |
| Reporting biases | 21 | Present assessments of risk of bias due to missing results (arising from reporting biases) for each synthesis assessed. | NA |
| Certainty of evidence | 22 | Present assessments of certainty (or confidence) in the body of evidence for each outcome assessed. | Table S4, Page 7 |
| **DISCUSSION** | | |  |
| Discussion | 23a | Provide a general interpretation of the results in the context of other evidence. | 7 |
|  | 23b | Discuss any limitations of the evidence included in the review. | 8 |
|  | 23c | Discuss any limitations of the review processes used. | 8 |
|  | 23d | Discuss implications of the results for practice, policy, and future research. | 8 |
| **OTHER INFORMATION** | | |  |
| Registration and protocol | 24a | Provide registration information for the review, including register name and registration number, or state that the review was not registered. | 3 |
|  | 24b | Indicate where the review protocol can be accessed, or state that a protocol was not prepared. | 3 |
|  | 24c | Describe and explain any amendments to information provided at registration or in the protocol. | NA |
| Support | 25 | Describe sources of financial or non-financial support for the review, and the role of the funders or sponsors in the review. | 8 |
| Competing interests | 26 | Declare any competing interests of review authors. | 8 |
| Availability of data, code and other materials | 27 | Report which of the following are publicly available and where they can be found: template data collection forms; data extracted from included studies; data used for all analyses; analytic code; any other materials used in the review. | 8 |

**Table S2.** Inclusion and Exclusion criteria

**Research Question:** What is are the impacts of PPI use on bone metabolism related outcomes?

| **Inclusion** | | **Exclusion** |
| --- | --- | --- |
| **Participants** | - General population | No restriction |
| **Intervention** | - Any type and dose of proton pump inhibitors (PPI) | Other anti-acidic drugs |
| **Comparator** | - Placebo or no drug | None |
| **Outcome** | - All type of fracture risks, Hip fracture, spine fracture, wrist fracture, any fracture - Osteoporosis - Bone mineral density changes - Hypomagnesemia - Osteointegration and failure of implants | Outcomes not related to bone metabolism |
| **Study Designs** | - Systematic review or Meta-analysis | Observational studies (cross sectional, cohort, case-control), Clinical trials, Letter to editor,  Commentaries,  Qualitative studies, Abstract only, Case series, case reports, reviews, Discussion papers, animal studies |
|  | - Geography-Global level - Date of Search- 16th of September 2023 |  |
|  | Published articles and preprints data in English | Unavailable full-text articles |

**Table S3. The adjusted search terms as per searched electronic databases [as of 16.09.2023]**

| **Database** | **No** | **Search Query** | **Results** |
| --- | --- | --- | --- |
| **PubMed** | | | |
|  | **#1** | **("Proton pump inhibitor" OR "Proton pump inhibitors" OR PPIs OR PPI OR Omeprazole OR Esomeprazole OR Lansoprazole OR Pantoprazole OR Rabeprazole OR Dexlansoprazole) AND (Bone OR BMD OR osteoporosis OR fracture OR hypomagnesemia OR implant OR "osse*") AND ("systematic review" OR "meta-analysis")** | **145** |
|  | | | |
| **Embase** | | | |
|  | **#1** | **('proton pump inhibitor':ab,ti OR 'proton pump inhibitors':ab,ti OR ppis:ab,ti OR ppi:ab,ti OR omeprazole:ab,ti OR esomeprazole:ab,ti OR lansoprazole:ab,ti OR pantoprazole:ab,ti OR rabeprazole:ab,ti OR dexlansoprazole:ab,ti) AND (bone:ab,ti OR bmd:ab,ti OR osteoporosis:ab,ti OR fracture:ab,ti OR hypomagnesemia:ab,ti OR implant:ab,ti OR 'osse*':ab,ti) AND ('systematic review':ab,ti OR 'meta-analysis':ab,ti)** | **91** |
| **Cochrane** | | | |
|  | **#1** | **(“Proton pump inhibitor” OR “Proton pump inhibitors” OR PPIs OR PPI OR Omeprazole OR Esomeprazole OR Lansoprazole OR Pantoprazole OR Rabeprazole OR Dexlansoprazole) AND (Bone OR BMD OR osteoporosis OR fracture OR hypomagnesemia OR implant OR “osse*”) in All Text** | **61** |
| **Web of science** | | | |
|  | **#1** | **(“Proton pump inhibitor” OR “Proton pump inhibitors” OR PPIs OR PPI OR Omeprazole OR Esomeprazole OR Lansoprazole OR Pantoprazole OR Rabeprazole OR Dexlansoprazole) AND (Bone OR BMD OR osteoporosis OR fracture OR hypomagnesemia OR implant OR “osse*”) AND (“systematic review” OR “meta-analysis”) (Topic)** | **83** |

**Table S4.** GRADE assessment for the included reviews

| **PPI compared to No PPI in Bone metabolism** | | | | | | | | | | | |
| --- | --- | --- | --- | --- | --- | --- | --- | --- | --- | --- | --- |
| **Certainty assessment** | | | | | | | **Summary of findings** | | | | |
| **Participants (studies) Follow-up** | **Risk of bias** | **Inconsistency** | **Indirectness** | **Imprecision** | **Publication bias** | **Overall certainty of evidence** | **Study event rates (%)** | | **Relative effect (95% CI)** | **Anticipated absolute effects** | |
|  |  |  |  |  |  |  | **With No PPI** | **With PPI** |  | **Risk with No PPI** | **Risk difference with PPI** |
| **Any Fracture (assessed with: RR)** | | | | | | | | | | | |
| 0 (14 observational studies) | not serious | serious^a^ | not serious | not serious | none | ⨁◯◯◯ Very low |  |  | **RR 1.20** (1.09 to 1.36) | 0 per 1,000 | **1 fewer per 1,000** (from 1 fewer to 1 fewer) |
| **Fracture risk in children (assessed with: RR)** | | | | | | | | | | | |
| 0 (5 observational studies) | not serious | not serious | not serious | not serious | none | ⨁⨁◯◯ Low |  |  | **RR 1.12** (1.07 to 1.17) | 0 per 1,000 | **1 fewer per 1,000** (from 1 fewer to 1 fewer) |
| **Fracture risk in Young adults** | | | | | | | | | | | |
| 0 (2 observational studies) | not serious | serious^a^ | not serious | serious^b^ | none | ⨁◯◯◯ Very low |  |  | **RR 0.98** (0.31 to 1.65) | 0 per 1,000 | **1 fewer per 1,000** (from 2 fewer to 0 fewer) |
| **Hip Fracture (assessed with: RR)** | | | | | | | | | | | |
| 0 (26 observational studies) | not serious | serious^a^ | not serious | not serious | none | ⨁◯◯◯ Very low |  |  | **RR 1.20** (1.13 to 1.27) | 0 per 1,000 | **1 fewer per 1,000** (from 1 fewer to 1 fewer) |
| **Hip fracture in post menopausal women (assessed with: RR)** | | | | | | | | | | | |
| 0 (5 observational studies) | not serious | not serious | not serious | serious^b^ | none | ⨁◯◯◯ Very low |  |  | **RR 1.23** (0.80 to 1.58) | 0 per 1,000 | **1 fewer per 1,000** (from 2 fewer to 1 fewer) |
| **Spine fracture (assessed with: RR)** | | | | | | | | | | | |
| 0 (6 observational studies) | not serious | not serious | not serious | not serious | none | ⨁⨁◯◯ Low |  |  | **RR 1.40** (1.08 to 1.64) | 0 per 1,000 | **1 fewer per 1,000** (from 2 fewer to 1 fewer) |
| **Wrist fracture** | | | | | | | | | | | |
| 0 (3 observational studies) | not serious | serious^a^ | not serious | serious^b^ | none | ⨁◯◯◯ Very low |  |  | **RR 1.08** (0.70 to 1.44) | 0 per 1,000 | **1 fewer per 1,000** (from 1 fewer to 1 fewer) |
| **Osteoporosis (assessed with: RR)** | | | | | | | | | | | |
| 0 (6 observational studies) | not serious | serious^a^ | not serious | serious^b^ | none | ⨁◯◯◯ Very low |  |  | **RR 1.22** (0.98 to 1.46) | 0 per 1,000 | **1 fewer per 1,000** (from 1 fewer to 1 fewer) |
| **Bone mineral density (all) (assessed with: MD)** | | | | | | | | | | | |
| 0 (7 observational studies) | not serious | serious^a^ | not serious | serious^b^ | none | ⨁◯◯◯ Very low |  |  | - | The mean bone mineral density (all) was **0** MD | MD **0.025 MD higher** (0.001 higher to 0.05 higher) |
| **Hypomagnesemia (assessed with: OR)** | | | | | | | | | | | |
| 0 (12 observational studies) | not serious | serious^a^ | not serious | not serious | none | ⨁◯◯◯ Very low |  |  | **OR 1.70** (1.33 to 2.19) | 0 per 1,000 | **2 fewer per 1,000** (from 2 fewer to 1 fewer) |
| **Implant failure (assessed with: RR)** | | | | | | | | | | | |
| 0 (4 RCTs) | not serious | serious^a^ | not serious | serious^c^ | strong association | ⨁⨁⨁◯ Moderate |  |  | **RR 3.16** (1.25 to 7.94) | 0 per 1,000 | **3 fewer per 1,000** (from 8 fewer to 1 fewer) |

**CI:** confidence interval; **MD:** mean difference; **OR:** odds ratio; **RR:** risk ratio

#### Explanations

a. High heterogeneity

b. Confidence limits overlap null effect

c. Wide confidence levels

**Table S5.** Quality assessment using JBI critical appraisal checklist for systematic reviews and research synthesis.

| **STUDY** | **Q1** | **Q2** | **Q3** | **Q4** | **Q5** | **Q6** | **Q7** | **Q8** | **Q9** | **Q10** | **Q11** |
| --- | --- | --- | --- | --- | --- | --- | --- | --- | --- | --- | --- |
| Chappuis et al. 2021 | Yes | Unclear | Yes | Yes | Yes | Unclear | Unclear | Yes | Unclear | Unclear | Unclear |
| Srinutta al. 2016 | Yes | Yes | Yes | Yes | Yes | Unclear | Unclear | Yes | Yes | Unclear | Unclear |
| Aghaloo et al. 2014 | Yes | Unclear | Yes | Yes | Unclear | No | Unclear | Yes | Unclear | Unclear | Unclear |
| Vinnakota et al. 2022 | Yes | Yes | Yes | Yes | Yes | Unclear | No | Yes | Yes | No | Unclear |
| Aleraij et al. 2020 | Yes | Unclear | Yes | Yes | Yes | No | No | Yes | Yes | Unclear | Unclear |
| Ngamruengphong et al. 2011 | Yes | Yes | Yes | Yes | Unclear | Yes | Yes | Unclear | Yes | No | Unclear |
| Ye et al. 2011 | Yes | Yes | Yes | Yes | Unclear | Unclear | No | Yes | Unclear | Unclear | Unclear |
| Nassar et al. 2018 | Yes | Y Unclear | Unclear | Yes | Unclear | Unclear | No | Yes | Yes | Unclear | Unclear |
| Liu et al. 2019 | Yes | Yes | Yes | Yes | Unclear | Yes | No | Yes | Yes | Unclear | Unclear |
| Da Maia et al. 2022 | Yes | Yes | Yes | Yes | Yes | Yes | No | Unclear | No | No | No |
| Poly et al. 2019 | No | Yes | Unclear | Yes | Unclear | Unclear | Yes | Unclear | Yes | Yes | No |
| Hussain et al. 2018 | Yes | Yes | Yes | Yes | Yes | Unclear | Unclear | Unclear | yes | Unclear | Unclear |
| Zhang et al. 2022 | Yes | Yes | Yes | Yes | Yes | Yes | Yes | Unclear | Yes | Yes | Yes |
| et al. |  |  |  |  |  |  |  |  |  |  |  |
| Verma et al. 2022 | Unclear | Yes | Yes | Yes | Yes | Yes | Unclear | Unclear | Yes | Yes | Yes |
| Yu et al. 2011 | Yes | Yes | Yes | Yes | Yes | Yes | Unclear | Unclear | Yes | Yes | Yes |
| Yang et al. 2022 | Yes | Yes | Yes | Yes | Yes | Yes | Yes | No | Yes | Yes | No |
| Mortensen et al. 2020 | Yes | Yes | Yes | Yes | Yes | Yes | No | Yes | Yes | Yes | No |
| Islam et al. 2018 | Yes | Yes | Yes | Yes | Yes | Yes | Yes | Yes | Yes | Yes | No |
| Eom et al. 2011 | Yes | Yes | Yes | Yes | Yes | Yes | Yes | Unclear | Yes | Yes | Yes |
| Kwok et al. 2011 | Yes | Y Unclear | Unclear | Yes | Unclear | Unclear | No | Yes | Yes | Unclear | Unclear |
| Fan 2017 | Yes | Yes | Yes | Yes | Yes | Unclear | Unclear | Unclear | yes | Unclear | Unclear |
| Li 2021 | Yes | Yes | Yes | Yes | Yes | Yes | Yes | Unclear | Yes | Yes | Yes |
| Cai 2015 | Yes | Yes | Yes | Yes | Unclear | Yes | No | Yes | Yes | Unclear | Unclear |
| Vestergaard 2019 | Yes | Yes | Yes | Yes | Yes | Yes | No | Unclear | No | No | No |
| Aggarwal 2019 | Yes | Yes | Yes | Yes | Yes | Unclear | Unclear | Unclear | yes | Unclear | Unclear |
| Heidelbaugh 2009 | Yes | Yes | Yes | Yes | Yes | Yes | Yes | Unclear | Yes | Yes | Yes |

**Forest plots**


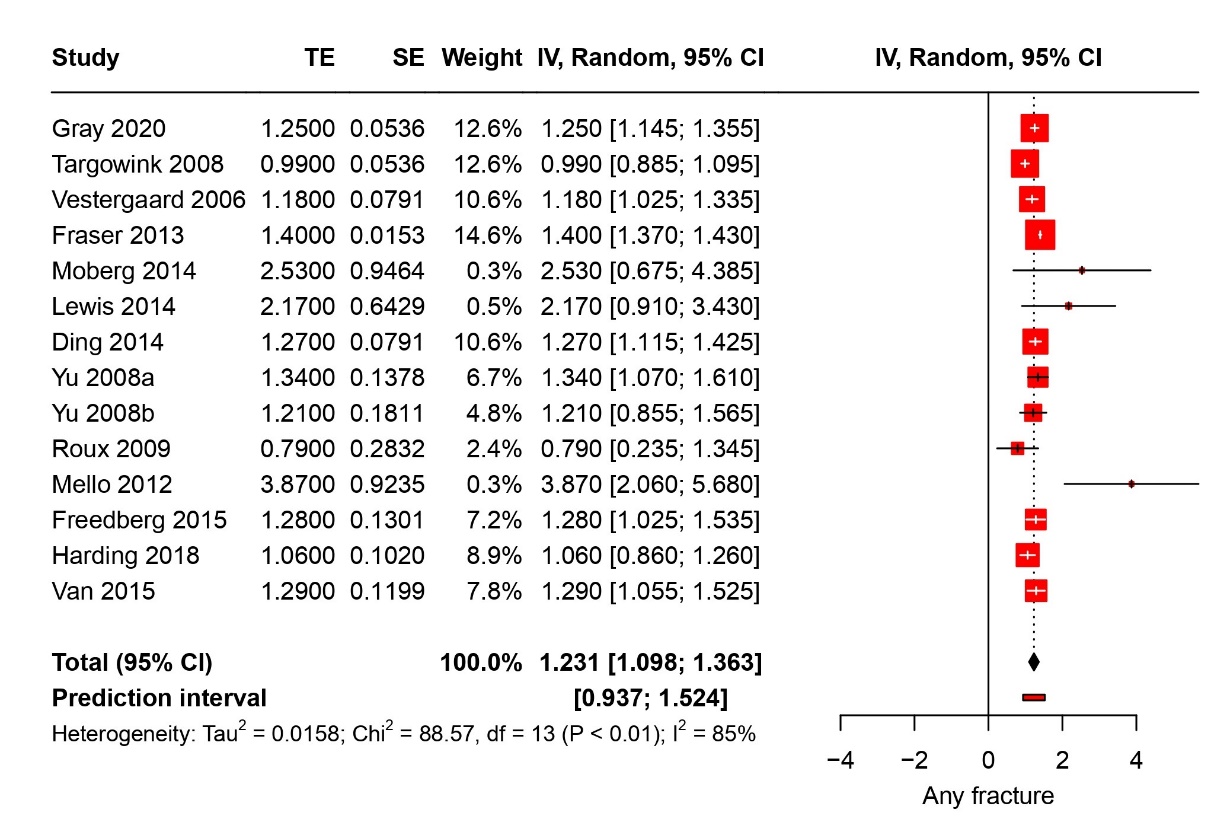


**Risk of Any fracture**


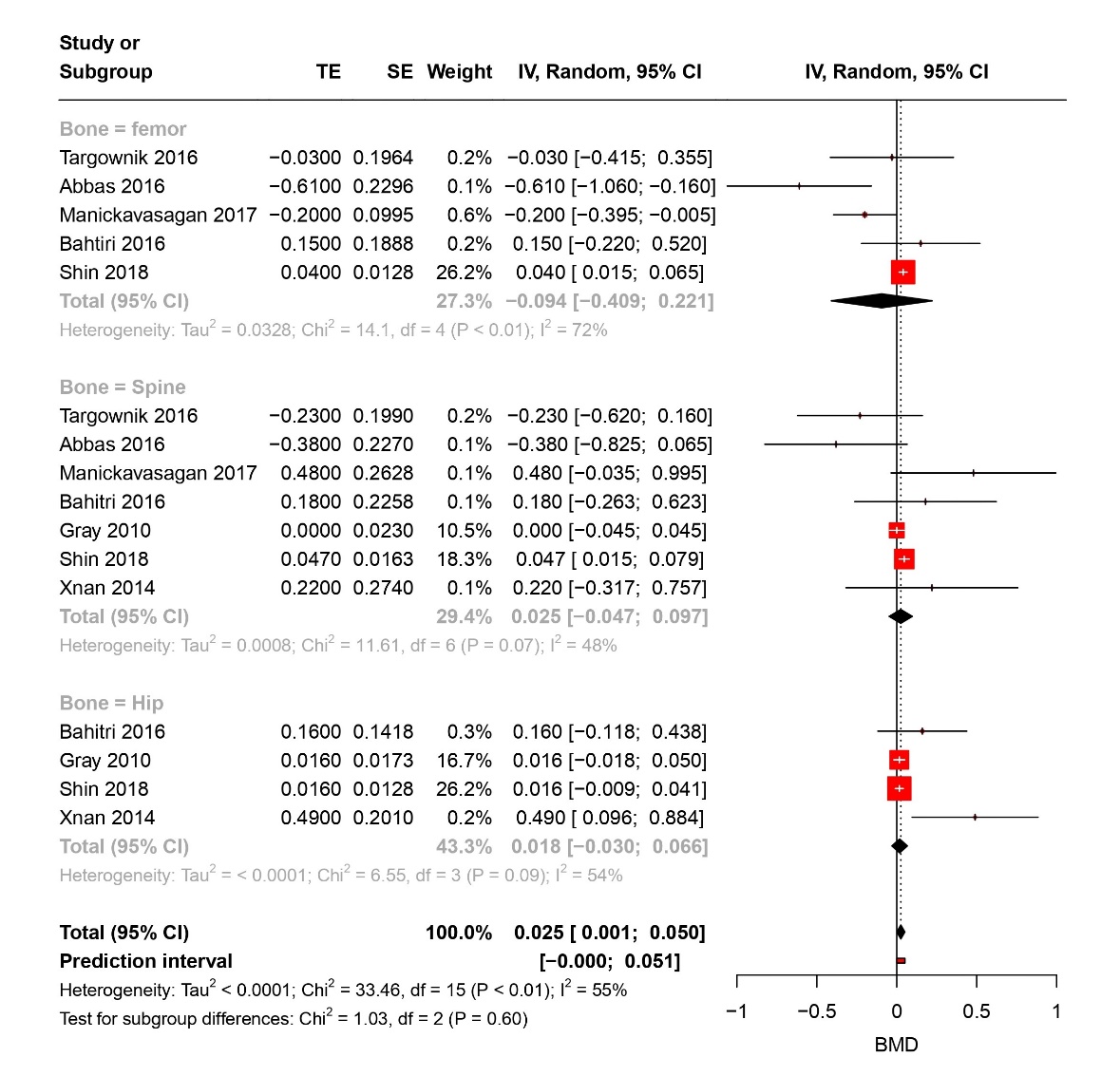


**Change in Mean BMD**


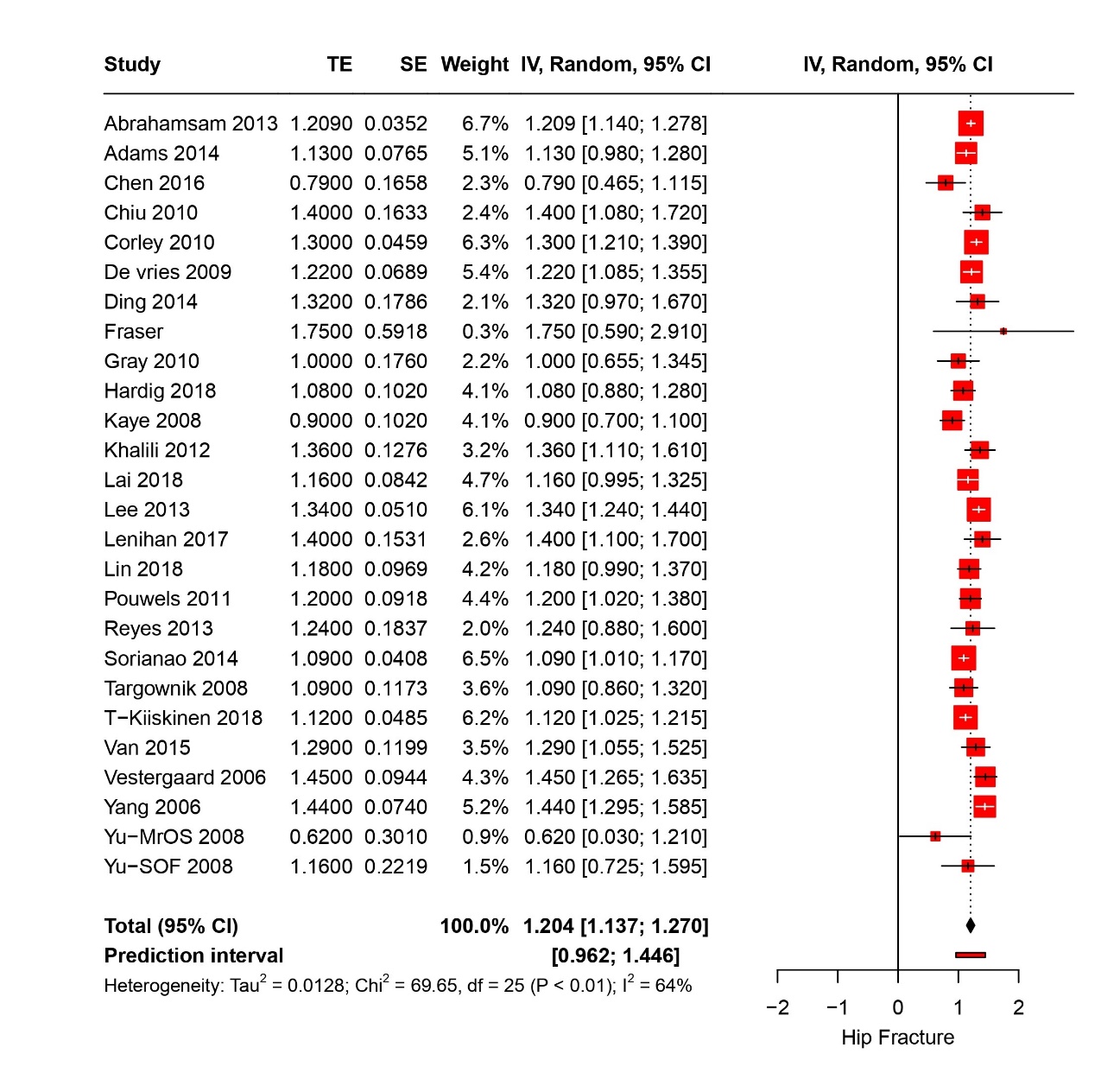


**Risk of Hip fracture**


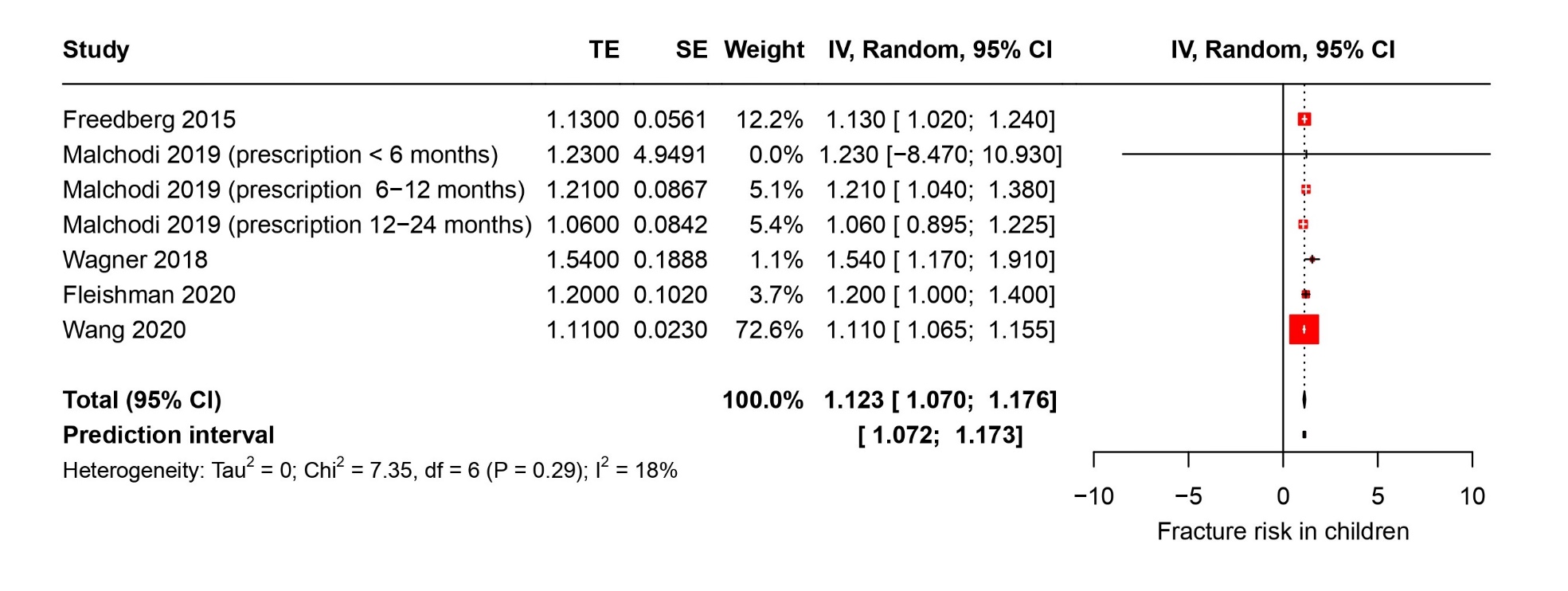


**Fracture risk in children**


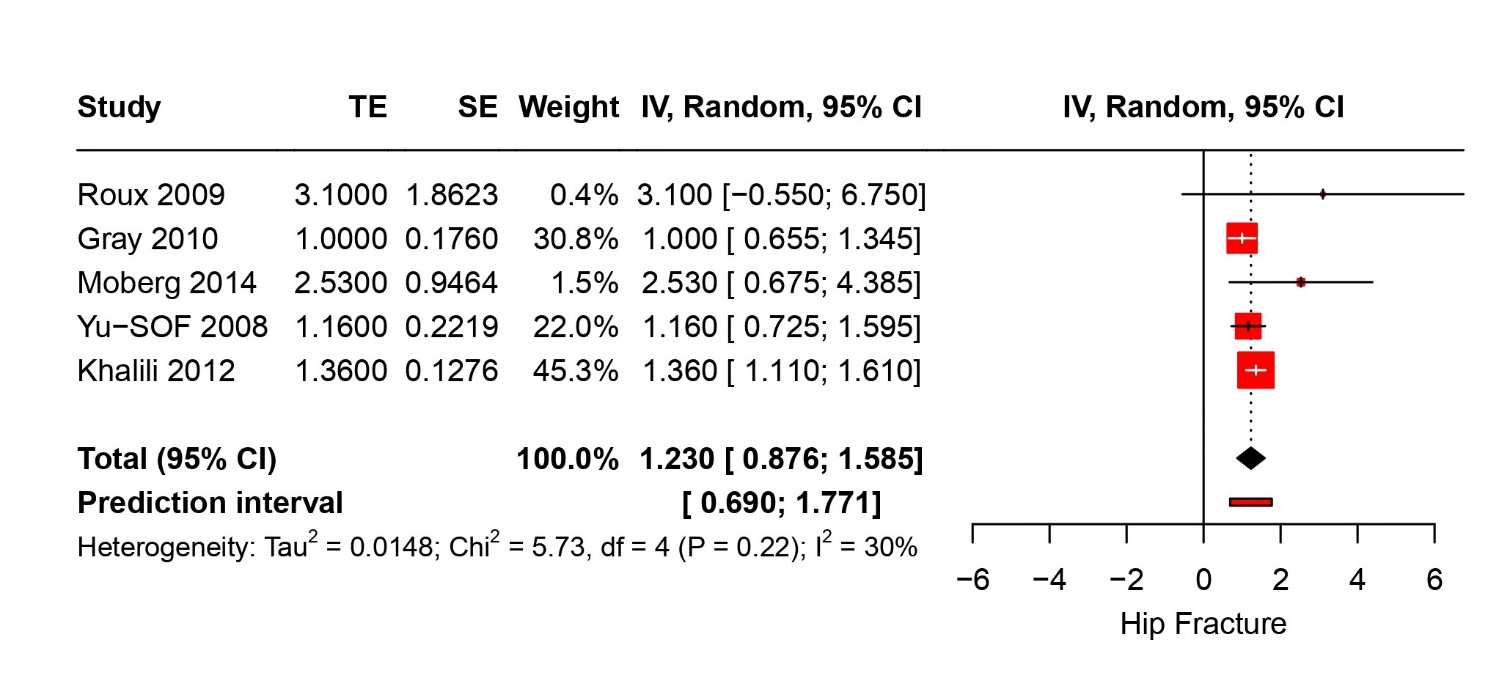


**Risk of Hip fracture in post-menopausal women**


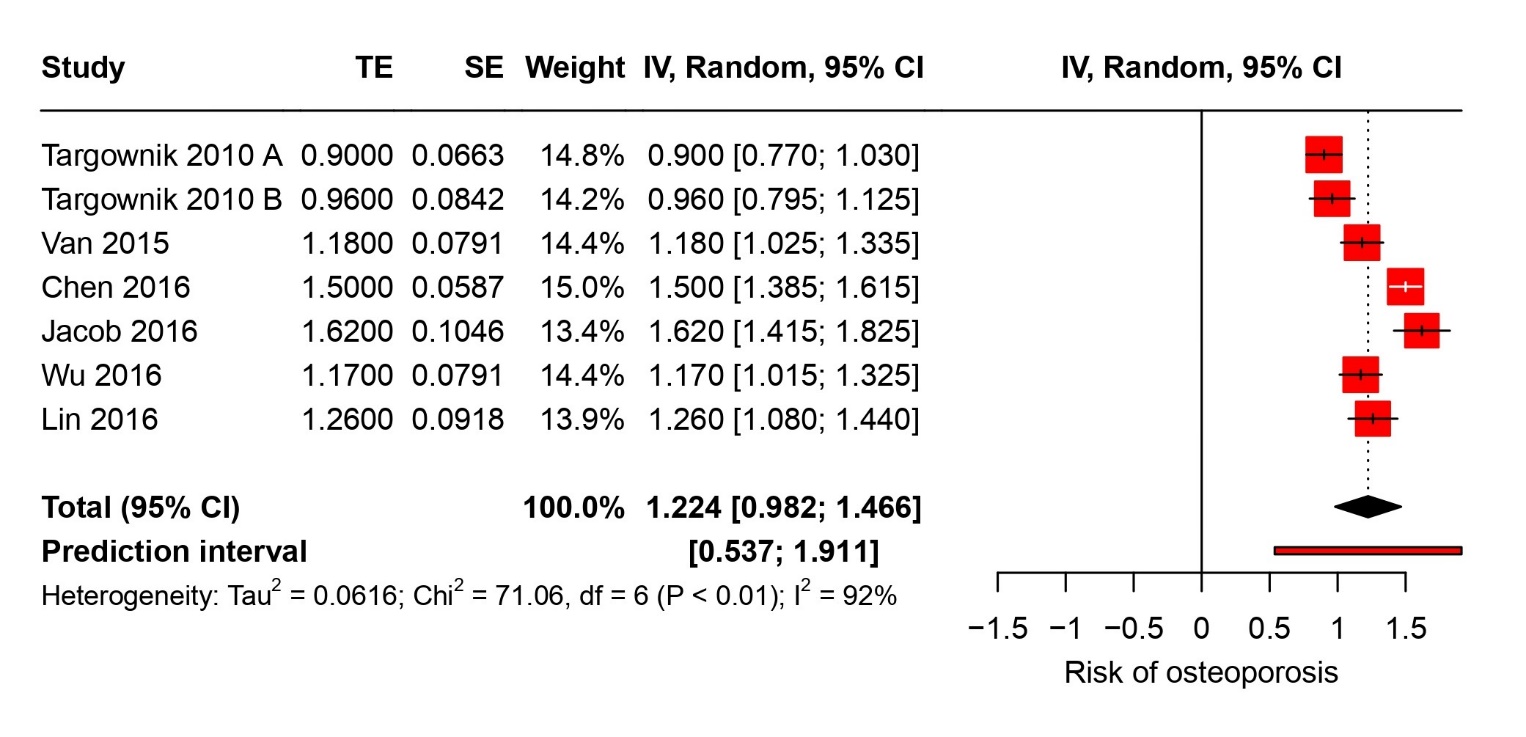


**Risk of osteoporosis**

**Funnel plots**


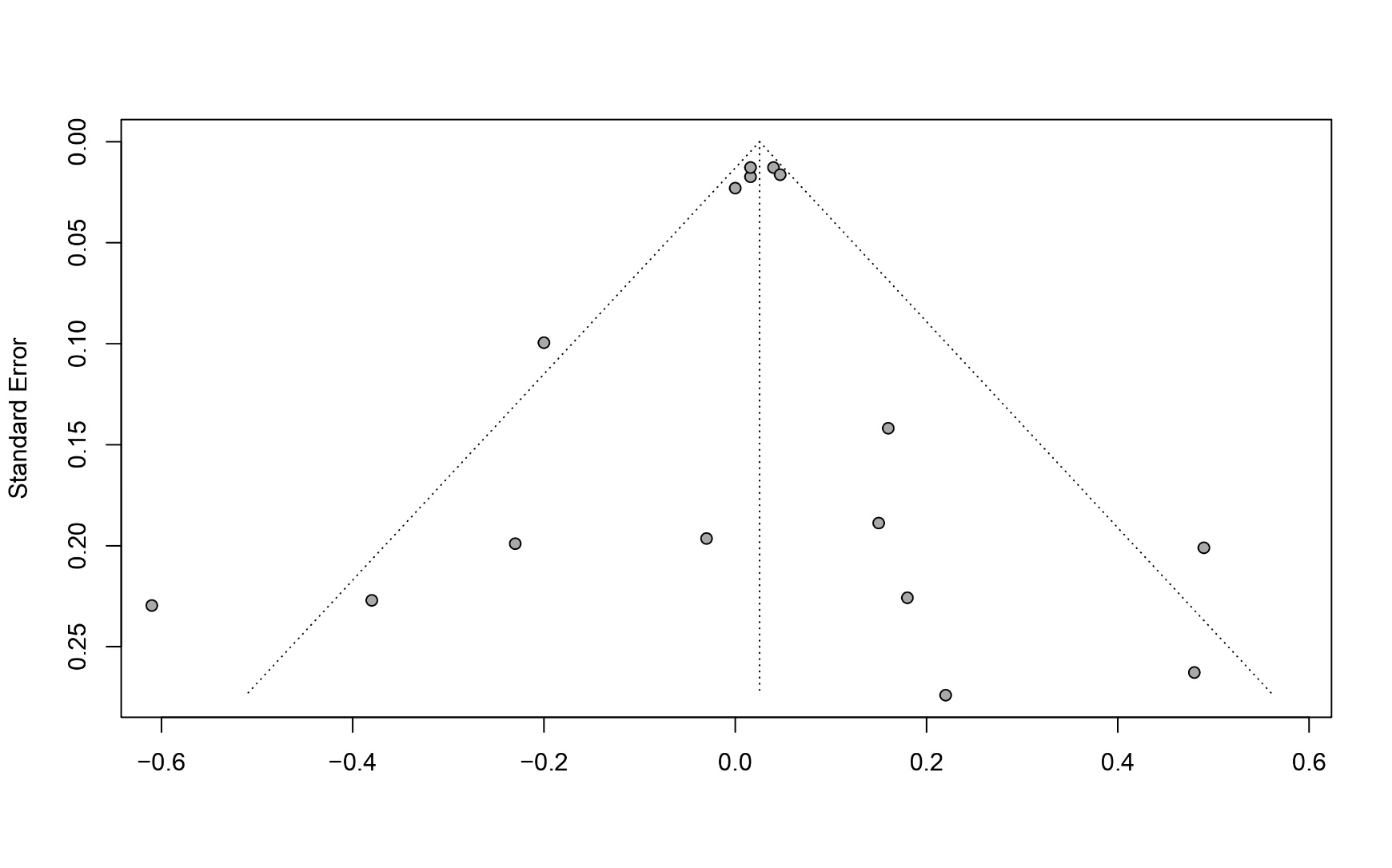


**Funnel plot of BMD**


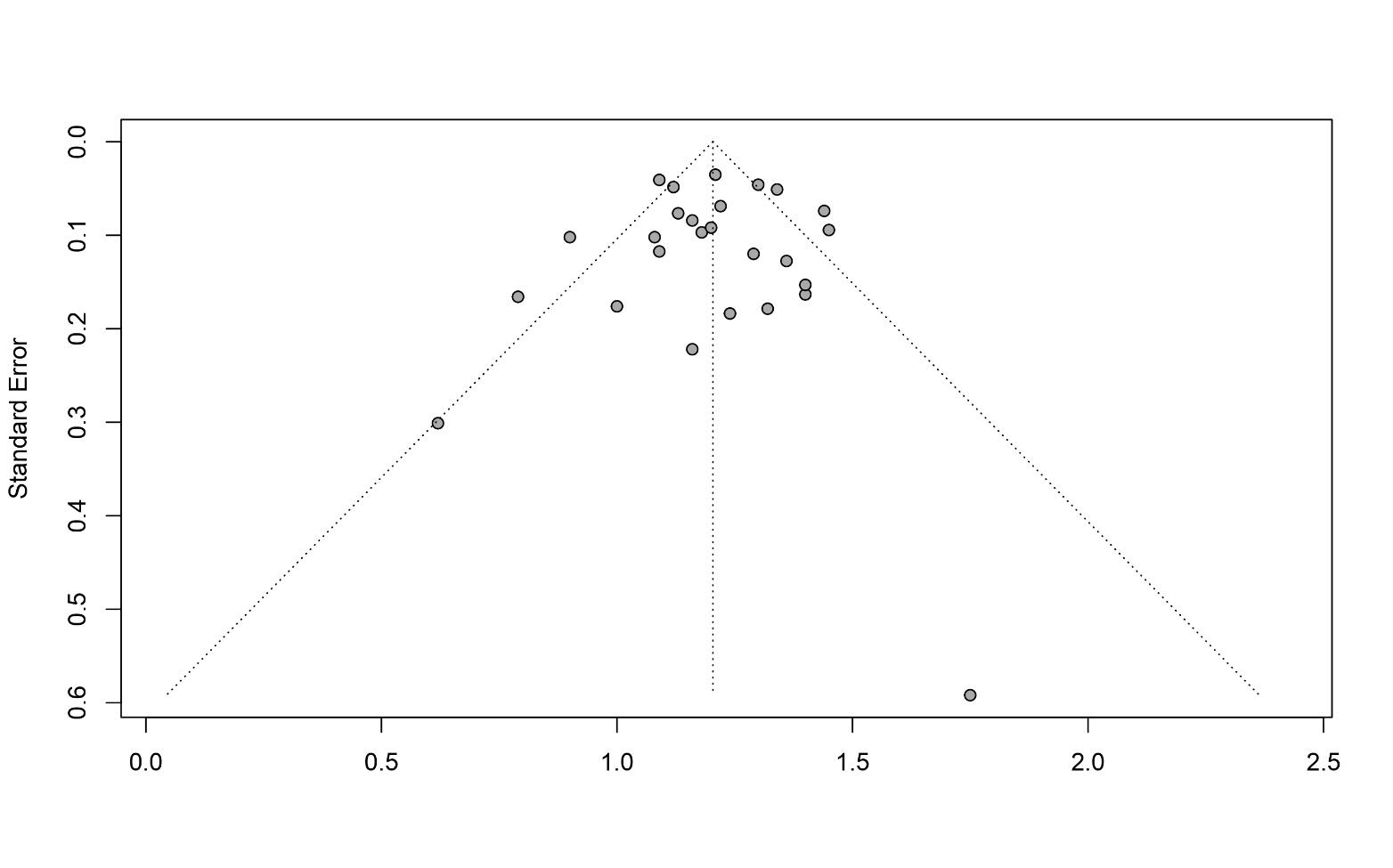


**Funnel plot of Hip fracture**

1. Aggarwal A, Sharma M, Maisnam I, Ghosh S, Aggarwal S, Bhattacharya S, et al. Drug-induced bone disorders: a systematic review. Indian Journal of Rheumatology. 2019;14(Suppl 1):S44-S51.

2. Aghaloo T, Pi-Anfruns J, Moshaverinia A, Sim D, Grogan T, Hadaya D. The Effects of Systemic Diseases and Medications on Implant Osseointegration: A Systematic Review. International Journal of Oral & Maxillofacial Implants. 2019;34.

3. Aleraij S, Alhowti S, Ferwana M, Abdulmajeed I, Mutawwam IM. Effect of proton pump inhibitors on bone mineral density: A systematic review and meta-analysis of observational studies. Bone reports. 2020;13:100732.

4. Cai D, Feng W, Jiang Q. Acid-suppressive medications and risk of fracture: an updated meta-analysis. International journal of clinical and experimental medicine. 2015;8(6):8893.

5. Chappuis V, Avila‐Ortiz G, Araújo MG, Monje A. Medication‐related dental implant failure: systematic review and meta‐analysis. Clinical oral implants research. 2018;29:55-68.

6. da Maia TF, de Camargo BG, Pereira ME, de Oliveira CS, Guiloski IC. Increased Risk of Fractures and Use of Proton Pump Inhibitors in Menopausal Women: A Systematic Review and Meta-Analysis. International Journal of Environmental Research and Public Health. 2022;19(20):13501.

7. Eom C-S, Park SM, Myung S-K, Yun JM, Ahn J-S. Use of acid-suppressive drugs and risk of fracture: a meta-analysis of observational studies. The Annals of Family Medicine. 2011;9(3):257-67.

8. Fan X-D, Ayom MIN, Sun W-G, Yin P-P, Wang X-Y, Jia A, et al. An updated meta-analysis: the effect of proton pump inhibitor on risk of osteoporosis and fracture. Int J Clin Exp Med. 2017;10(11):15680-95.

9. Heidelbaugh JJ, Goldberg KL, Inadomi JM. Adverse risks associated with proton pump inhibitors: a systematic review. Gastroenterology & Hepatology. 2009;5(10):725.

10. Hussain S, Siddiqui AN, Habib A, Hussain MS, Najmi AK. Proton pump inhibitors’ use and risk of hip fracture: a systematic review and meta-analysis. Rheumatology international. 2018;38(11):1999-2014.

11. Islam MM, Poly TN, Walther BA, Dubey NK, Ningrum DNA, Shabbir S-A, et al. Adverse outcomes of long-term use of proton pump inhibitors: a systematic review and meta-analysis. European journal of gastroenterology & hepatology. 2018;30(12):1395-405.

12. Kwok CS, Yeong JK-Y, Loke YK. Meta-analysis: risk of fractures with acid-suppressing medication. Bone. 2011;48(4):768-76.

13. Li J, Xie X, Liu W, Gu F, Zhang K, Su Z, et al. Acid-suppressive drugs and risk of fracture in children and young adults: a meta-analysis of observational studies. Frontiers in Pharmacology. 2021;12:712939.

14. Liu J, Li X, Fan L, Yang J, Wang J, Sun J, et al. Proton pump inhibitors therapy and risk of bone diseases: An update meta-analysis. Life sciences. 2019;218:213-23.

15. Mortensen SJ, Mohamadi A, Wright CL, Chan JJ, Weaver MJ, von Keudell A, et al. Medications as a risk factor for fragility hip fractures: a systematic review and meta-analysis. Calcified tissue international. 2020;107:1-9.

16. Nassar Y, Richter S. Proton-pump inhibitor use and fracture risk: an updated systematic review and meta-analysis. Journal of bone metabolism. 2018;25(3):141-51.

17. Ngamruengphong S, Leontiadis GI, Radhi S, Dentino A, Nugent K. Proton pump inhibitors and risk of fracture: a systematic review and meta-analysis of observational studies. Official journal of the American College of Gastroenterology| ACG. 2011;106(7):1209-18.

18. Poly T, Islam M, Yang H-C, Wu C, Li Y-C. Proton pump inhibitors and risk of hip fracture: a meta-analysis of observational studies. Osteoporosis International. 2019;30:103-14.

19. Srinutta T, Chewcharat A, Takkavatakarn K, Praditpornsilpa K, Eiam-Ong S, Jaber BL, et al. Proton pump inhibitors and hypomagnesemia: A meta-analysis of observational studies. Medicine. 2019;98(44):e17788.

20. Verma V. Do proton pump inhibitors affect the biomechanical efficiency of implant?-a systematic review. Journal of Oral Biology and Craniofacial Research. 2022.

21. Vestergaard P. Drugs causing bone loss. Bone Regulators and Osteoporosis Therapy. 2020:475-97.

22. Vinnakota DN, Kamatham R. Effect of proton pump inhibitors on dental implants: A systematic review and meta-analysis. The Journal of the Indian Prosthodontic Society. 2020;20(3):228.

23. Yang J, Zhou T-j, Yang J, Bao D-n. Use of acid-suppressive drugs and risk of fracture in children and young adults: a meta-analysis of observational studies. European Journal of Clinical Pharmacology. 2022:1-9.

24. Ye X, Liu H, Wu C, Qin Y, Zang J, Gao Q, et al. Proton pump inhibitors therapy and risk of hip fracture: a systematic review and meta-analysis. European journal of gastroenterology & hepatology. 2011;23(9):794-800.

25. Elaine WY, Bauer SR, Bain PA, Bauer DC. Proton pump inhibitors and risk of fractures: a meta-analysis of 11 international studies. The American journal of medicine. 2011;124(6):519-26.

26. Zhang Y, Deng D, Zhang R, Yi J, Dong J, Sha L. Relationship between proton pump inhibitors and adverse effects in hemodialysis patients: a systematic review and meta-analysis. Kidney and Blood Pressure Research. 2022;47(9):545-55.

27. Zhou B, Huang Y, Li H, Sun W, Liu J. Proton-pump inhibitors and risk of fractures: an update meta-analysis. Osteoporosis international. 2016;27:339-47.
